# Suppression of TLR4-MyD88 signaling pathway attenuated chronic mechanical pain in a rat model of endometriosis

**DOI:** 10.1186/s12974-020-02066-y

**Published:** 2021-03-05

**Authors:** Wenliang Su, Huan Cui, Danning Wu, Jiawen Yu, Lulu Ma, Xiuhua Zhang, Yuguang Huang, Chao Ma

**Affiliations:** 1 Department of Anesthesiology, Peking Union Medical College Hospital, Chinese Academy of Medical Sciences and Peking Union Medical College, Beijing, China; 2 Department of Human Anatomy, Histology and Embryology, Institute of Basic Medical Sciences, Neuroscience Center, Chinese Academy of Medical Sciences, School of Basic Medicine, Peking Union Medical College, Beijing, China

**Keywords:** Endometriosis, Pain, HMGB1, Toll-like receptor 4, Dorsal root ganglion, Spinal dorsal horn

## Abstract

**Background:**

As a classic innate immunity pathway, Toll-like receptor 4 (TLR4) signaling has been intensively investigated for its function of pathogen recognition. The receptor is located not only on immune cells but also on sensory neurons and spinal glia. Recent studies revealed the involvement of neuronal TLR4 in different types of pain. However, the specific role of TLR4 signaling in the pain symptom of endometriosis (EM) remains obscure.

**Methods:**

The rat endometriosis model was established by transplanting uterine horn tissue into gastrocnemius. Western blotting and/or immunofluorescent staining were applied to detect high mobility group box 1 (HMGB1), TLR4, myeloid differentiation factor-88 adaptor protein (MyD88), and nuclear factor kappa-B-p65 (NF-κB-p65) expression, as well as the activation of astrocyte and microglia. The antagonist of TLR4 (LPS-RS-Ultra, LRU) and MyD88 homodimerization inhibitory peptide (MIP) were intrathecally administrated to assess the behavioral effects of blocking TLR4 signaling on endometriosis-related pain.

**Results:**

Mechanical hyperalgesia was observed at the graft site, while HMGB1 was upregulated in the implanted uterine tissue, dorsal root ganglion (DRG), and spinal dorsal horn (SDH). Compared with sham group, upregulated TLR4, MyD88, and phosphorylated NF-κB-p65 were detected in the DRG and SDH in EM rats. The activation of astrocytes and microglia in the SDH was also confirmed in EM rats. Intrathecal application of LRU and MIP alleviated mechanical pain on the graft site of EM rats, with decreased phosphorylation of NF-κB-p65 in the DRG and reduced activation of glia in the SDH.

**Conclusions:**

HMGB1-TLR4-MyD88 signaling pathway in the DRG and SDH may involve in endometriosis-related hyperpathia. Blockade of TLR4 and MyD88 might serve as a potential treatment for pain in endometriosis.

**Supplementary Information:**

The online version contains supplementary material available at 10.1186/s12974-020-02066-y.

## Introduction

Endometriosis (EM), defined as the emergence of endometrial tissue outside the uterus, is a common and costly estrogen-dependent disorder, affecting about 176 million women worldwide [1]. The hallmark symptom of EM is chronic pain, mostly occurring around the ectopic uterine tissue [2, 3]. Classically, the available treatments included pain medications, hormones, surgery, and fertility treatment [4–7]. However, the outcomes were unsatisfying or with intolerable side effects, such as tissue lesions and the maladjustment of estrogen [8–10]. Identifying the pathogenesis of pain in EM and developing effective treatments remain necessary.

High mobility group box 1 (HMGB1), a potent pro-inflammatory mediator, is an endogenous ligand of TLRs, including TLR2, TLR4, and TLR5, which might lead to allodynia through activating TLR signaling [11]. In addition, HMGB1 served as a potential indicator of fibrosis in ectopic uterine tissue [12], which also had been identified as a promising and admissible biomarker in plasma for EM. Sensory neurons in the DRG, especially the nociceptors, could be facilitated by HMGB1 in an advanced glycosylation end product-specific receptor (RAGE) and transient receptor potential vanilloid-1 (TRPV1) dependent manner [11, 13, 14]. The spinal HMGB1 was also sufficient and necessary for both local and generalized hypersensitivity in the partial infraorbital nerve transection model and contributed to analgesic tolerance under the condition of chronic morphine usage [15, 16].

Toll-like receptors (TLRs) function as sensors for external pathogens in innate immunity. TLR4 protects the host cells by recognizing external pathogens and adjusting downstream inflammatory cascades [17]. Recent studies suggested that TLR4 was expressed in small-diameter primary sensory neurons, which might regulate nociceptive sensation like itch and pain [18–20]. The combination of HMGB1 and TLR4 induced the recruitment of myeloid differentiation factor-88 adaptor protein (MyD88) and NF-κB-depended transcriptional process in sensory neurons and glial cells, which was associated with multiple types of pain [21–25]. Nerve injury also elicited intensive neuroinflammation in the DRG through the HMGB1-TLR4-MyD88 signaling pathway, featured by the prominent production of TNF-α and IL-1β [26–28]. In the SDH, TLR4-positive astrocytes and microglial cells evoked the central sensitization and promoted the induction and persistence of pain by producing multiple cytokines [29, 30].

Considering the essential role of HMGB1 in EM’s pathogenesis, we hypothesized that the HMGB1-TLR4-MyD88 signaling pathway in the DRG and SDH was activated, which promoted the development of pain in EM. The suppression of TLR4 and Myd88 could alleviate mechanical pain around the ectopic uterine tissue. We aimed to reveal the changes of HMGB1-TLR4-MyD88 signaling pathway in DRG and SDH in a rat EM model, and the behavioral effects of blocking TLR4 and MyD88 on EM-related pain were also explored. This study might provide potential therapeutic strategies for the pain symptom in EM.

## Methods

### Animals

Adult female Sprague Dawley rats (180–220 g) were purchased from HFK Bioscience Co., Ltd, Beijing, China. All were housed in a controlled environment (room temperature 21 ± 4 °C, lights on 07:00–19:00 h, 4–5 rat per cage) with food and water ad libitum. All experimental procedures were approved by the Institutional Animal Care and Use Committee in Chinese Academy of Medical Sciences, Institute of Basic Medical Sciences.

### The rat endometriosis model

The rat model of endometriosis was performed as previously reported [3, 31, 32]. Adult female rats were pre-medicated with intraperitoneal sodium pentobarbital injection (40 mg/kg) under aseptic conditions. After laparotomy, the abdominal cavity was exposed, and the right uterine horn was identified and isolated. One centimeter segment was excised and placed in a Petri dish containing 0.9% NaCl. The excised uterine horn was opened longitudinally, and a piece of full-thickness (3 × 3 mm) uterine tissue was implanted onto the right gastrocnemius muscle by single stitches. The sham surgery followed the same procedure, but the implant sutured to the gastrocnemius muscle was replaced by a 3 × 3 mm peritoneal fat instead of uterine tissue.

### Drug delivery

To complete the intrathecal administration, A PE10 conduit (18 cm in length) was implanted intrathecally under anesthesia with inhaled isoflurane (2.5%), according to previous reports [33]. Briefly, after making a longitudinal incision (1 cm) between the L4 and L5 vertebrae, a fine needle was inserted through the dura mater, and then the PE 10 conduit was placed rostrally in the subarachnoid space. The proper intrathecal placement was confirmed by checking for a sudden flicking of tail or hindlimb as well as cerebrospinal fluid outflow. The tip of the conduit was fixed at the cervical region of the rats through a subcutaneous tunnel. Before rats were performed with the model of endometriosis as above, they were injected with 2% lidocaine (20 μL) through the conduit. Only rats whose hind legs were paralyzed in 30 s after injection and did not recover within 30 min were included in the experiment.

LPS-RS-Ultra (LRU, InvivoGen, San Diego, CA, USA; dissolved in endotoxin-free water), a TLR4-specific antagonist, was administered through the PE10 conduit (20 μg in 20 μL). MyD88 homodimerization inhibitory peptide (MIP, Novus Biologicals, CO, USA), a MyD88 inhibitory peptide or control peptide (Novus Biologicals, CO, USA) dissolved in 20 μL PBS (500 μM) was administered according to previous studies [34].

### Behavioral assay

Muscular tenderness was tested by an electronic pressure measuring instrument, as described previously [31, 32]. Rats were cautiously put into a cylindrical acrylic holder with an adjustable baffle. It was intended for easy exposure of hind limbs and measurement of mechanical pain threshold at the implantation site, namely the belly of the gastrocnemius muscle. Before each behavior test, rats were acclimated in the cylindrical acrylic holder for 30 min. The pressure was applied gradually to the gastrocnemius through a 7-mm-diameter probe; the force of which was assessed by the Compact Digital Force Gauge (Wagner, FDX 10, Shanghai Yuyan Instruments Co., Ltd., China). The mechanical threshold was defined as the force in Newtons, and an acute withdrawal of hind leg was considered as a positive response. The baseline mechanical pain threshold was defined as the mean of 3 readings taken at 5-min intervals before surgery.

### Western blot assay

After deeply anesthetized with sodium pentobarbital (40 mg/kg) and transcardially perfused with sterile PBS, the L4–L6 spinal dorsal horn (SDH) and dorsal root ganglions (DRGs) were excised and quick-frozen in liquid nitrogen. The whole samples were homogenized in T-PER Tissue Protein Extraction Reagent (Thermo Fisher Scientific, USA) containing Protein Phosphatase Inhibitor (Solarbio, Beijing, China) and phosphatase inhibitor Cocktail (CWbio, Beijing, China). The lysates were then homogenized and centrifuged (12000×*g* for 15 min at 4 °C). The supernatants were collected and denatured, and then separated by SDS-polyacrylamide gel electrophoresis gels and transferred to a PVDF membrane (Thermo Fisher Scientific, USA). The membranes were blocked with 5% (w/v) BSA (A8020, Bioss, Beijing, China) for 1 h at room temperature and incubated with primary antibodies overnight at 4 °C, followed by incubation with corresponding secondary antibody for 1 h at room temperature (Table 1 lists the primary and secondary antibodies used for western blot analysis). The bands were scanned with Tanon 5800 Luminescent Imaging Workstation (Tanon Science & Technology Co., Ltd. Shanghai, China) by High-sig ECL Western Blotting Substrate (Tanon, China). The band intensity was measured by Image J software (National Institutes of Health, Bethesda, MD, USA). The ratio of target protein versus β-actin in each sample, reflected by the ratio of band intensity, was used to compare the relative content of target protein among different samples.

**Table 1:** List of primary and secondary antibodies used for western blotting analysis

Antibody	Host	Company	Catalog number	Dilution	Incubation conditions
HMGB1	Rabbit	Cell signaling technology	#6893	1:1000	Overnight 4 °C
MyD88	Rabbit	Abcam	ab2064	1:500	Overnight 4 °C
TLR4	Rabbit	Invitrogen	48-2300	1:1000	Overnight 4 °C
pNF-κB-p65	Mouse	Cell signaling technology	#13346	1:1000	Overnight 4 °C
β-actin	Mouse	Cell signaling technology	#3700	1:2000	Overnight 4 °C
Anti-rabbit IgG horseradish peroxidase (HRP)	Goat	ZSGB-BIO	ZDR-5306	1:3000	1 h RT
Anti-mouse IgG horseradish peroxidase (HRP)	Goat	ZSGB-BIO	ZDR-5307	1:3000	1 h RT

### Immunofluorescence staining

Immunofluorescence was performed as Liu et al. described [34]. Rats were pre-medicated with intraperitoneal sodium pentobarbital injection (40 mg/kg) under aseptic condition and then transcardially perfused with sterile PBS followed by 4 °C paraformaldehyde (Sigma, USA). The L4–L6 spinal dorsal horn (SDH) and DRGs were collected, fixed in 4% paraformaldehyde overnight at 4 °C, and then dehydrated in 30% sucrose until the tissue settled to the bottom of the EP pipe at 4 °C. Later, tissues were embedded in OCT (Tissue-Tek) and serially sectioned in a cryostat (Leica 2000, Germany) into 15-μm-thickness slices. After permeabilized in 0.3% Triton X-100 for 15 min (DRGs) or 1 h (SDH) at 37 °C, the tissue sections were blocked with 5% donkey serum for 1 h, and then bound with the target primary antibodies at 4 °C overnight in a wet box. Corresponding secondary antibodies were incubated for 1 h at room temperature (Table 2 lists the primary and secondary antibodies used for immunofluorescence staining analysis). The slides were then washed in PBS and coverslipped by Mounting Medium with DAPI (ZSJB-Bio, Beijing, China). Images were captured by laser confocal microscopic imaging system (FV1000 and Olympus FluoView software, Olympus, Japan). The quantification for immunofluorescence staining referred to previous studies [20, 34–36]. Briefly, at least 12 fields from 3 randomly selected sections on each ganglion were examined. More than 100 neuronal somata with nucleus profiles for each condition were counted, and the percentiles of immunopositive neurons were reported. According to the cross-sectional areas (CSA) of soma, neurons were classified as small- (CSA < 636 μm^2^), medium- (CSA 637–1431 μm^2^), and large-sized neurons (CSA > 1431 μm^2^). Three non-adjacent spinal cord sections were randomly selected from the spinal cord segment (L3–L5) for each rat. Three rats were included for each group. The intensity of GFAP and IBA1 staining was measured with Image J.

**Table 2:** List of primary and secondary antibodies used for immunofluorescence staining

Antibody	Host	Company	Catalog ID	Dilution	Incubation conditions
HMGB1	Rabbit	Cell Signaling Technology	#6893	1:200	Overnight 4 °C
MyD88	Rabbit	Abcam	ab2064	1:400	Overnight 4 °C
TLR4	Rabbit	Invitrogen	48-2300	1:400	Overnight 4 °C
TRPV1	Guinea pig	Abcam	ab10295	1:400	Overnight 4 °C
PGP9.5	Guinea pig	Abcam	ab10410	1:400	Overnight 4 °C
pNF-κB-p65	Rabbit	Abcam	ab86299	1:400	Overnight 4 °C
GFAP	Chichen	Abcam	ab4674	1:1000	Overnight 4 °C
CGRP	Goat	Abcam	ab36001	1:3000	Overnight 4 °C
IBA1	Goat	Abcam	ab5076	1:500	Overnight 4 °C
NeuN	Mouse	Abcam	ab104224	1:1000	Overnight 4 °C
Anti-rabbit IgG Alexa Fluor 488	Donkey	Jackson ImmunoResearch	711-545-152	1:400	1 h RT
Anti-Guinea pig IgG Alexa Fluor 594	Donkey	Jackson ImmunoResearch	706-585-148	1:400	1 h RT
Isolectin GS-IB4 Alexa Fluor™ 594 Conjugate		Invitrogen	I21413	1:400	1 h RT
Anti-goat IgG Alexa Fluor 488	Donkey	Abcam	ab150129	1:400	1 h RT
Anti-goat IgG Alexa Fluor 594	Donkey	Jackson ImmunoResearch	705-585-147	1:400	1 h RT

### Statistical analysis

Data were presented as group mean and its standard error (mean ± SEM). Shapiro-Wilk test was applied to determine the normality for parametric test. Student’s *t* test was used to examine differences between two groups, while one-way analysis of variance (ANOVA) followed by the Bonferroni post hoc test was used to examine the difference between multiple groups. For the behavioral test, two-way repeated measures ANOVA was used. In the two-way ANOVA tests, one factor was the time point, and the other was the treatment of the rats (receiving EM surgery or Sham; receiving LRU or Vehicle). If the differences were significant, post hoc Bonferroni’s test was applied to compare values at different time points. Categorical data was presented as numbers and percentages and compared by chi-square tests. And all statistical analyses were performed in GraphPad Prism for Windows version 7.0 (GraphPad Software, Inc., San Diego, CA, USA). A statistically significant difference was defined as a two-sided *p* value < 0.05.

## Results

### Construction of endometriosis in rats.

**Fig. 1:**
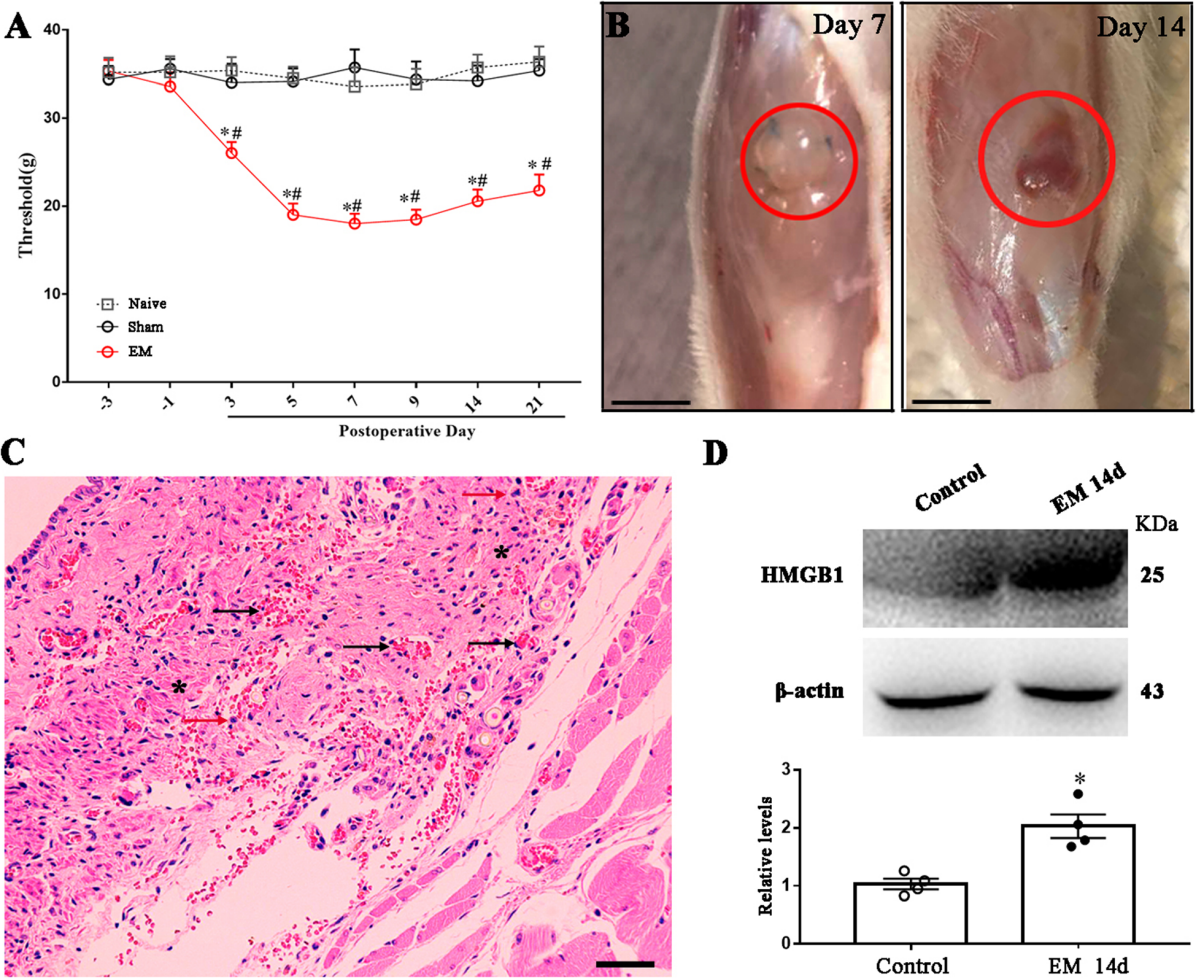
Effects of endometriosis on the pathologic, behavioral response, and HMGB1. **a** The time course of mechanical threshold at the graft site indicated EM-induced mechanical allodynia. *n* = 7, two-way ANOVA following Bonferroni’s post hoc test, ^*^p < 0.05, EM vs sham group; ^#^*p* < 0.05, EM vs naive group. **b** Representative dissection of transplant revealed cystic lesions with an amber fluid at postoperative day 7, and a rufous fluid at postoperative day 14. The red circle indicated the transplanted uterine. Scale bar 1 cm. **c** Representative HE staining at the lesion revealed stromal component contained fine capillary network filled with blood cells at postoperative day 14. Black arrows: congestion; red arrows: invasive immune cell; asterisk: fibroblastic proliferation. Scale bar 100 μm. **d** The representative protein bands of HMGB1 protein from control uterine and transplanted uterine on POD14 of EM model. *n* = 4, Student’s *t* test, ^*^p < 0.05

Surgical induction of endometriosis was successfully established in rats, and hyperalgesia was confirmed by behavioral tests. In order to exclude the pain induced by incision, behavioral tests were performed 3 days after surgery. Behavior test showed that rats with uterine grafts developed increased pressuring pain since the third day after operation compared with sham group (surgery with abdominal adipose tissue transplant) or naive group (no surgery) (Fig. 1a). On postoperative day (POD) 7 and 14, dissection of the transplanted tissue revealed cystic lesions with a reddish-brown to amber fluid in endometriosis rats, but not in sham-operated rats (Fig. 1b). HE staining showed that the transplanted uterus was integrated into surrounding muscles tightly with extensive fibroblastic proliferation and pervasive congestion (Fig. 1c). Consistent with the basic pathology and symptoms of endometriosis observed in patients clinically, we successfully established the endometriosis model in rats. To determine the expression of HMGB1 in transplant, western blotting was performed, and the data revealed that HMGB1 was upregulated in the transplant compared with the control uterus horn tissue (Fig. 1d).

### Upregulation of HMGB1 in nociceptive pathways after EM

**Fig. 2:**
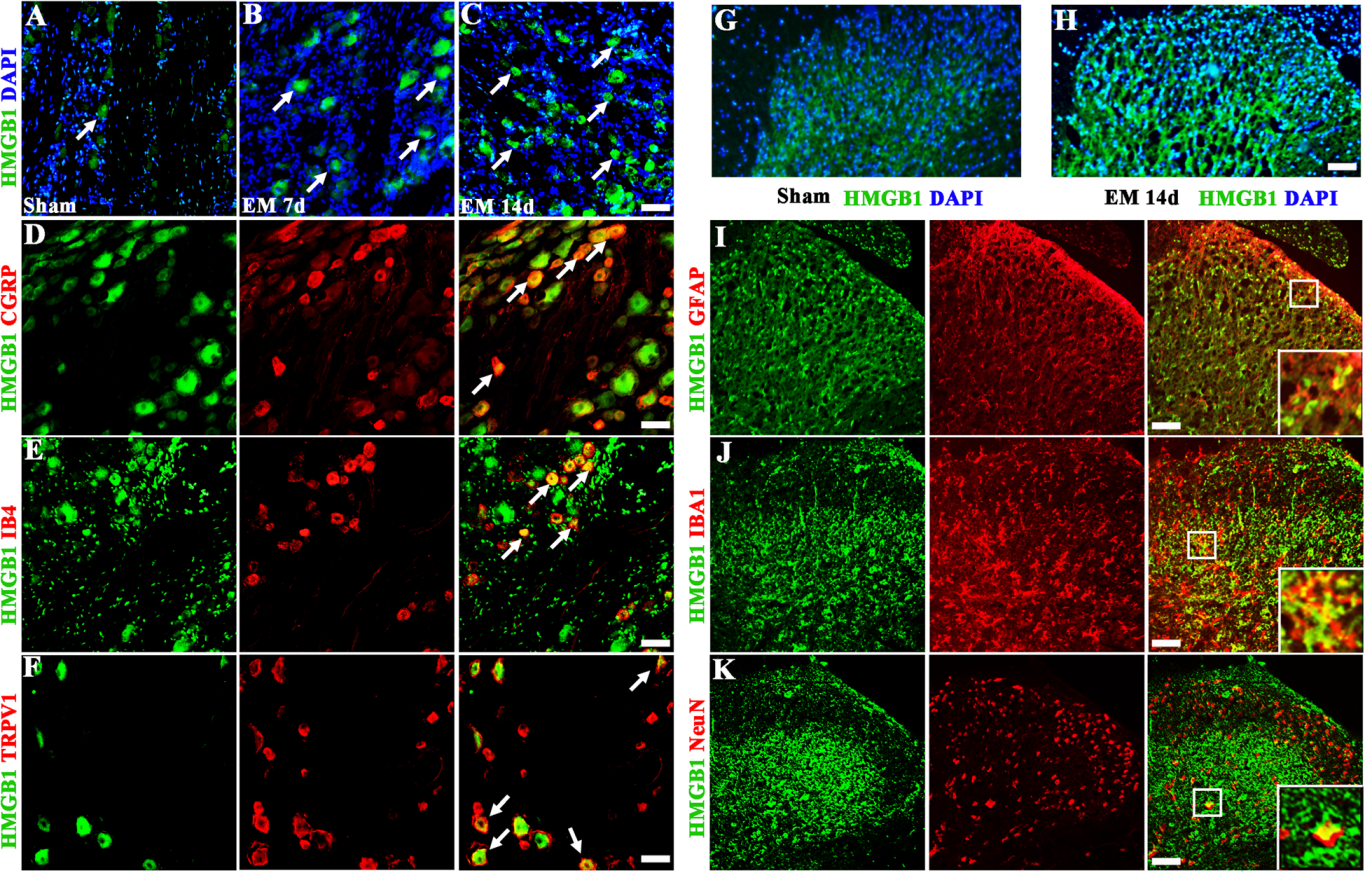
Endometriosis upregulated HMGB1 in the DRG and SDH. **a**–**c** Compared with the sham group, endometriosis upregulated cytoplasmic HMGB1 in sensory neurons in the dorsal root ganglion on POD7 and POD14 after surgery. Scale bar 50 μm. **d**–**f** On POD14 after endometriosis, double immunostaining showed colocalization of HMGB1 with nociceptor markers including CGRP, IB4, and TRPV1, respectively. Scale bar 50 μm. **g**, **h** Compared with the sham group, endometriosis upregulated HMGB1 in spinal dorsal horn on POD14 after surgery. Scale bar 50 μm. **i**–**k** On POD14 after endometriosis, immunofluorescence staining showed colocalization of HMGB1 with astrocytic marker GFAP, microglial marker IBA1 and neuronal marker NeuN. Scale bar 50 μm

The immunofluorescence results showed the expression of HMGB1 in sensory neurons. In addition, we found that the subcellular distribution of HMGB1 shifted from the nucleus to the cytoplasm, which indicated HMGB1 expression in its pro-inflammatory form. Compared with sham group (21.27%), cytoplasmic HMGB1 was upregulated on POD7 (40.36%) and POD14 (45.19%) for EM rats in the dorsal root ganglion (Fig. 2a–c and Fig. S1A). On POD14, we determined the subpopulation of HMGB1-positive neurons by double-immunofluorescence double staining technique. Summarily, we observed HMGB1 expressed in calcitonin gene-related peptide (CGRP)-positive (39.86%), non-peptidergic isolectin B4 (IB4)-positive (43.18%), and transient receptor potential vanilloid-1 (TRPV1)-positive (30.21%) neurons of DRGs (Fig. 2d–f and Fig. S1B).

According to the immunofluorescence results, the expression of HMGB1 was significantly upregulated in the SDH of EM rats compared with the sham group (Fig. 2g, h and Fig. S1C). And HMGB1 protein was mainly distributed in the superficial layers of the SDH. We also found that on POD14, the HMGB1 immunoreactivity was observed in astrocytes (co-localized with GFAP), microglial cells (co-localized with IBA1), and neuronal cells (co-localized with NeuN). These results suggested that EM induced the expression of HMGB1, peripherally and centrally, which might activate the downstream TLR4 signaling pathway (Fig. 2i–k).

### Endometriosis upregulated neuronal TLR4 in nociceptors

**Fig. 3:**
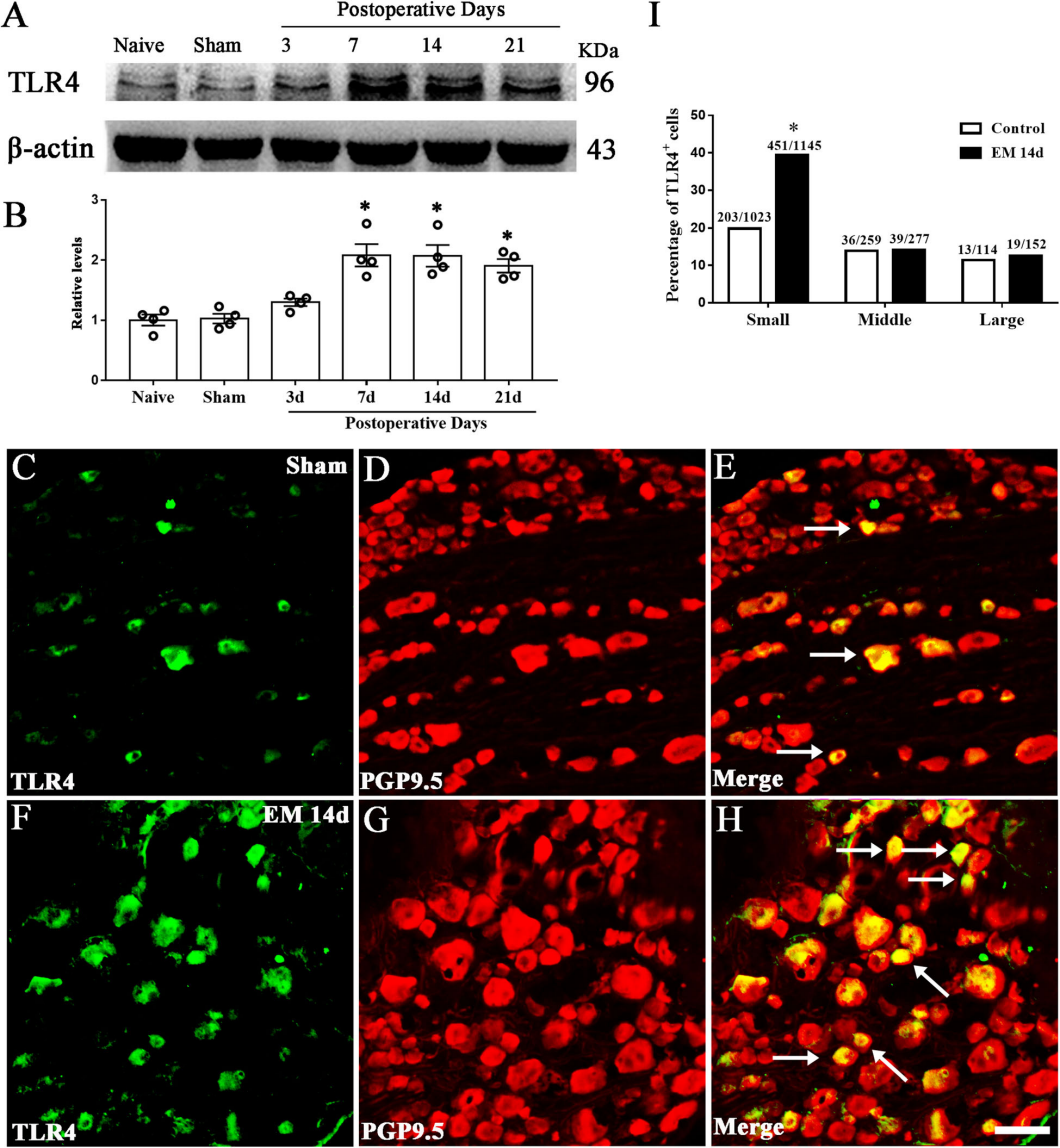
Endometriosis upregulated neuronal TLR4. **a** Western blot analyzed the time course of TLR4 expression in the DRG from naive, sham, 3 days, 7 days, 14 days, and 21 days after surgery. **b** *n* = 4, one-way ANOVA following Bonferroni's post-hoc test, *p < 0.05, versus naive and sham. **c**–**h** Immunofluorescence showed neuronal distribution of TLR4 in rat DRGs from sham and POD14 rats. Scale bar 50 μm. **i** TLR4 was expressed in the large-, medium-, and small-sized neurons in the dorsal root ganglion. Immunofluorescence staining showed a significantly elevated percentage of TLR4-immunopositive cells in small-sized DRG neurons of POD14 rats compared with that in sham rats. *n* > 100 neurons from 3 rats in each group, *p < 0.05, EM 14d versus sham, chi-square test

First, we determined the time course of TLR4 expression in DRG. The increased expression of TLR4 peaked at 7 days and maintained high throughout the testing periods in EM Group (Fig. 3a, b). Using double immunofluorescent staining, we found that TLR4 was expressed on DRG neurons that were immunopositive for neuron specific marker protein PGP9.5. Immunofluorescence showed TLR4 was upregulated in small-diameter neurons of DRG in EM group compared to sham group (sham 19.84%, 203/1023; EM 39.39%, 451/1145). However, there was no difference in TLR4 expression in middle-diameter (sham 13.90%, 36/259; EM 14.08%, 39/277) and large-diameter neurons (sham 11.40%, 13/114; EM 12.50%, 19/152), compared with the sham group (Fig. 3c, d).

**Fig. 4:**
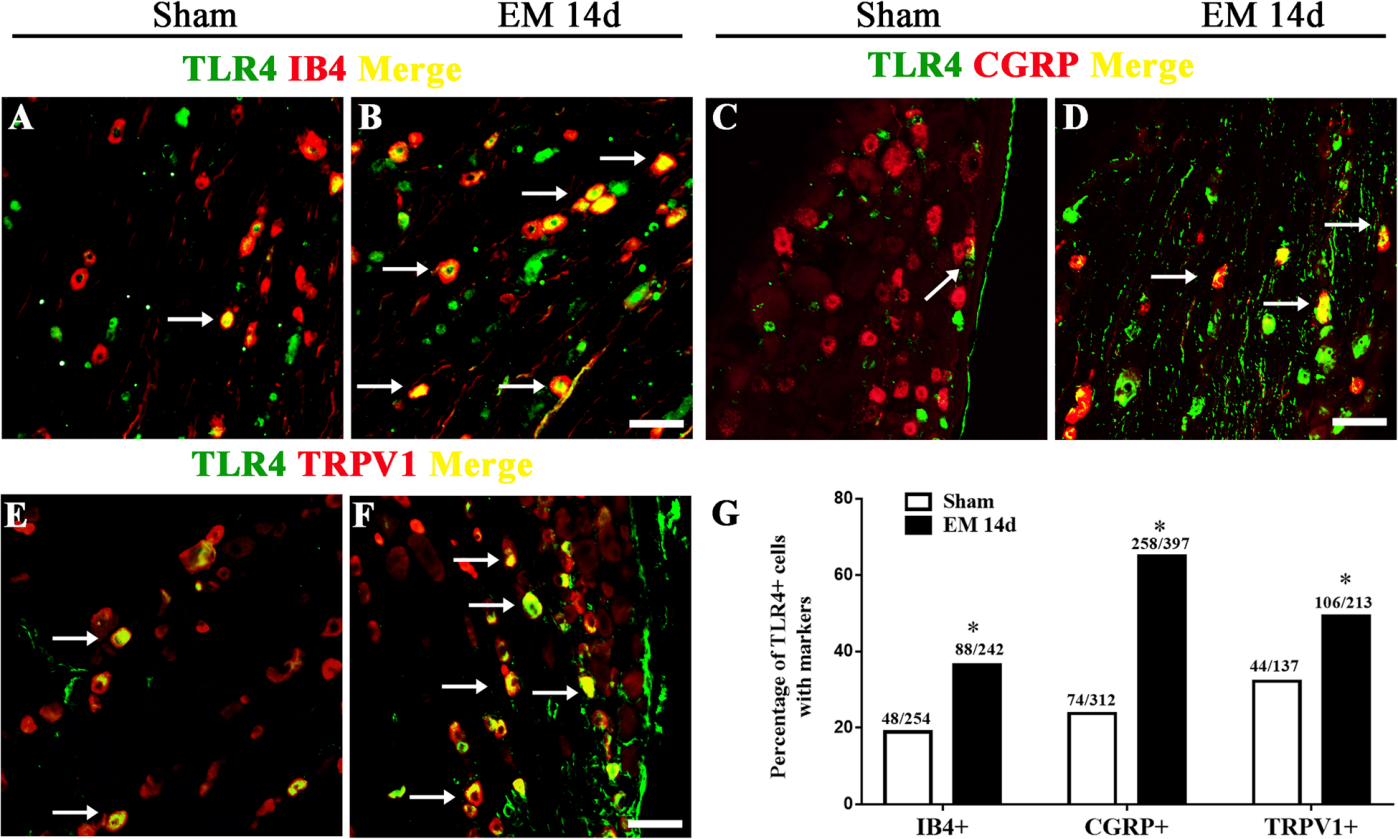
Endometriosis upregulated TLR4 in nociceptors. **a**–**h** Double immunostaining showed co-expression of TLR4 with nociceptive marker, CGRP, IB4, and TRPV1 in rat DRG from sham and EM 14d groups. Scale bar 50 μm. **i** Parentages of TLR4^+^ cells in neurons with nociceptive markers (IB4, CGRP, and TRPV1) were increased in the EM 14d group compared with that in sham group. *n* > 100 neurons from 3 rats in each group, *p < 0.05, EM 14d vs sham, chi-square test

In addition, to determine the characteristic of TLR4-positive sensory neurons, double immunofluorescence of TLR4 and nociceptive markers, including IB4, CGRP, and TRPV1 was performed. The TLR4 protein immunoreactivity was identified to be distributed in all three categories of nociceptor (Fig. 4a–f). Further comparison revealed that EM upregulated the expression of TLR4 in IB4^+^ (from 18.90 to 36.36%), CGRP^+^ (from 23.72 to 64.99%), and TRPV1^+^ (from 32.12 to 49.77%) nociceptors (Fig. 4g).

### Endometriosis upregulated neuronal MyD88 in nociceptors

**Fig. 5:**
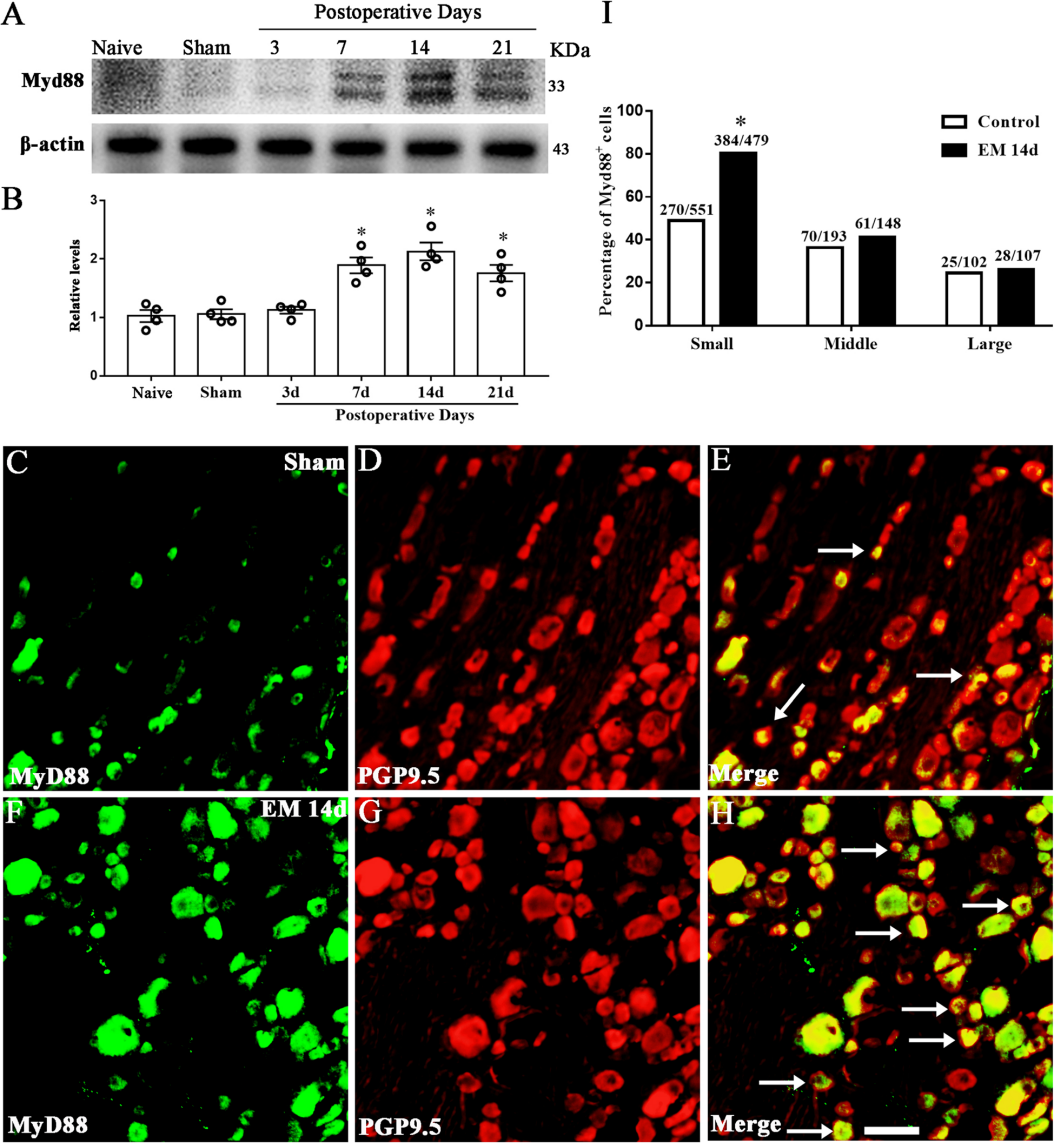
Endometriosis upregulated neuronal MyD88 in the dorsal root ganglion. **a** Western blot analyzed the time course of Myd88 expression in the DRG from naïve, sham, 3 days, 7 days, 14 days, and 21 days after surgery. **b** For the expression levels of Myd88, there was no significant difference between naïve, sham, and 3 days after surgery. On postoperative day 7, 14, and 21 group, Myd88 was upregulated in the DRG. *N* = 4, one-way ANOVA, *p < 0.05, versus naive and sham. **c**–**h** Immunofluorescence showed neuronal distribution of Myd88 in rat DRGs of sham and EM 14 days groups. Scale bar 50 μm. **i** Myd88 was expressed in the large-, medium-, and small-sized neurons in the dorsal root ganglion. Immunofluorescence staining showed a significantly higher percentage of Myd88-immunopositive cells in small-sized DRG neurons in EM 14d rats compared with that in sham rats. *n* > 100 neurons from 3 rats in each group, *p < 0.05, EM 14d vs sham, chi-square test

MyD88, the adaptor of TLR4, has also been found to participate in nociceptive sensation. In this study, we detected the expression of MyD88 in DRG and determined its changes in EM rats. Western blotting results showed that the level of MyD88 was gradually elevated, which peaked on POD14 and maintained high until POD21 (Fig. 5a, b). Immunofluorescence showed that MyD88 also located in sensory neurons, co-localizing with PGP 9.5 in the DRG. Compared with the sham group, the percentage of MyD88-positive neurons in DRG was elevated in the EM group (sham 43.14%, 365/846; EM 64.44%, 473/734), specifically in small-diameter neurons (sham 49.00%, 270/551; EM 80.17%, 384/479) (Fig. 5c, d).

**Fig. 6:**
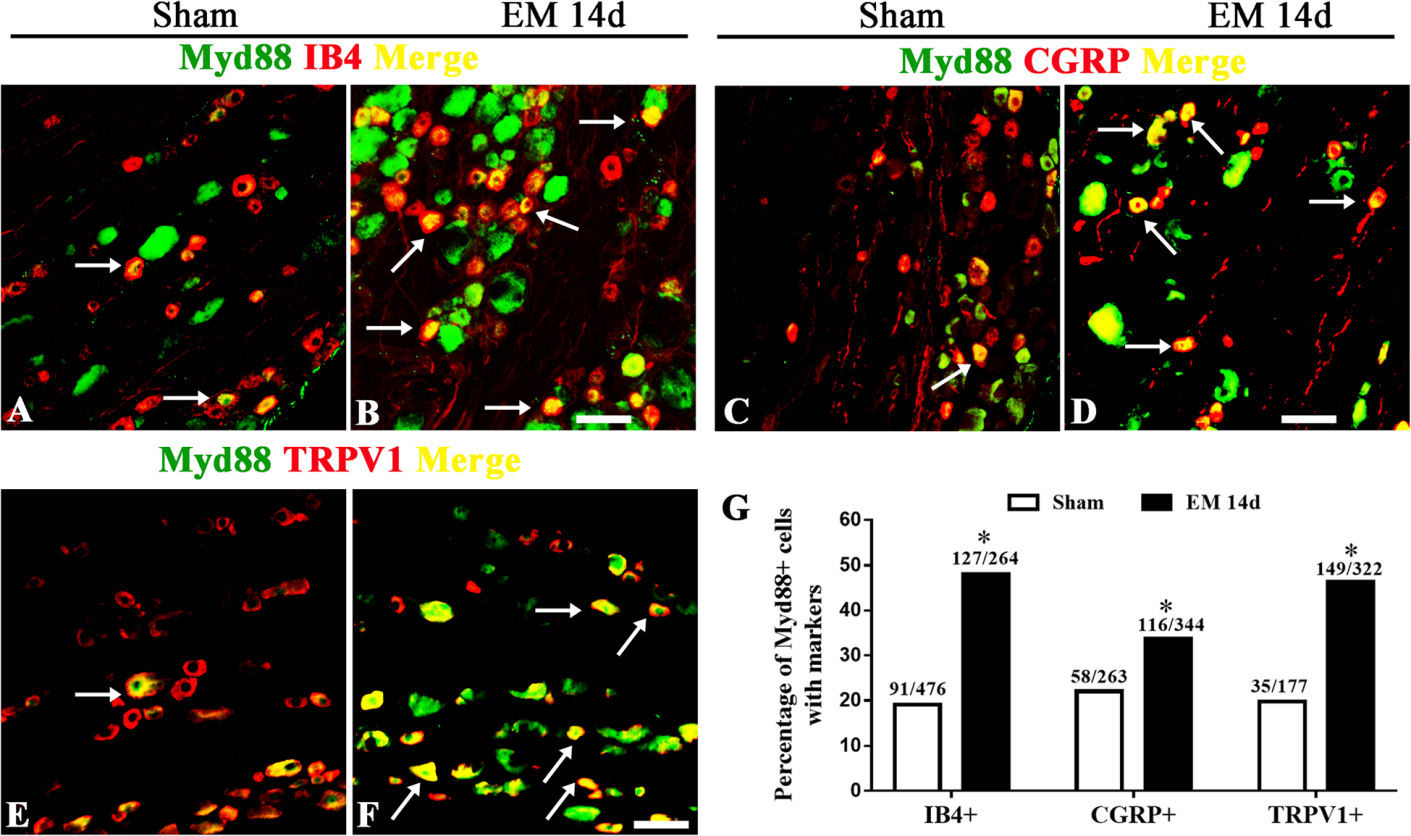
Endometriosis upregulated Myd88 in nociceptors. **a**–**f** Typical microscopic images of immunostaining for MyD88 co-expression with IB4, CGRP, and TRPV1 in rat DRG from sham and EM 14d groups. Scale bar 50 μm. **g** Parentages of Myd88^+^ cells in neurons with nociceptive markers (IB4, CGRP, and TRPV1) were increased in the EM 14d group compared with that in sham group. *n* > 100 neurons from 3 rats in each group, *p < 0.05, EM 14d vs sham, chi-square test

The nociceptive characteristic of MyD88^+^ sensory neurons was also determined by double immunofluorescence (Fig. 6a–f). The expression of MyD88 was detected in neurons with nociceptive markers including IB4 (sham 19.12%; EM 48.11%), CGRP (sham 22.05%; EM 33.72%) and TRPV1 (sham 19.77%; EM 46.27%), which was upregulated in EM (Fig. 6g). These results showed that TLR4-MyD88 signaling might be activated in nociceptors under the condition of EM.

### EM induced the activation of NF-κB in sensory neurons

**Fig. 7:**
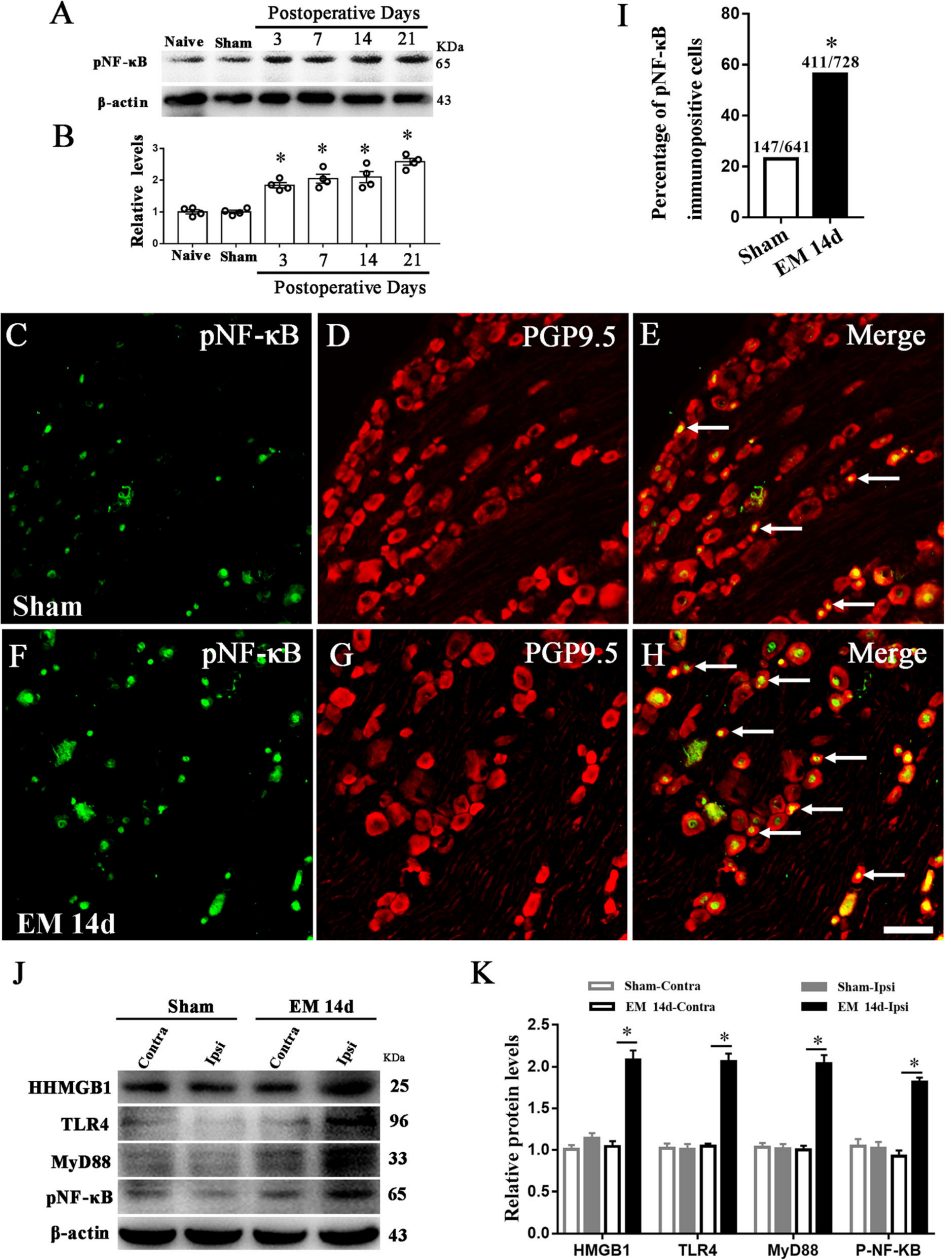
Endometriosis upregulated neuronal pNF-κB-p65 in the dorsal root ganglion. **a** Western blot analyzed the time course of pNF-κB-p65 expression in the DRG from naïve, sham, 3 days, 7 days, 14 days, and 21 days after EM surgery. **b** Western blot revealed that EM upregulated pNF-κB-p65 protein expression in L3–L5 DRGs. *N* = 4, one-way ANOVA, *p < 0.05, versus sham and naïve. **c**–**h** Immunofluorescence showed neuronal distribution of pNF-κB-p65 in rat DRG of sham and EM 14 days groups. Scale bar 50 μm. **i** Immunofluorescence staining showed a significantly higher percentage of pNF-κB-p65-immunopositive DRG neurons in EM 14d group compared with that in sham group. *n* > 100 neurons from 3 rats in each group, *p < 0.05, EM 14d vs sham, chi-square test. **j**, **k** Western blot showed that HMGB1, TLR4, MyD88, and pNF-κB-p65 were upregulated in the ipsilateral DRG compared with the contralateral DRG. *N* = 4, *p < 0.05, Student’s *t* test, EM 14d-Ipsi versus EM 14d-Contra

NF-κB is the transcription factor of the TLR4-MyD88 signaling pathway, which is activated by phosphorylation. Our western blotting results showed that activated phospho-NF-κB p65 increased gradually in DRG from POD3 to POD21, compared with naive and sham groups (Fig. 7a, b). Immunofluorescence showed an elevated expression of phospho-NF-κB p65 in sensory neurons in EM rats (sham 22.93%, 147/641; EM 56.46%, 411/728) (Fig. 7c–i). To eliminate the systemic effects on the DRG molecular changes, we compared the expression of HMGB1, TLR4, MyD88, and p-NF-κB in the contralateral and ipsilateral DRG by western blot. Our data showed that these proteins were upregulated in the ipsilateral DRG compared with the contralateral side (Fig. 7j–k). These results suggested that EM induced the activation of the TLR4/MyD88/NF-κB pathway in the ipsilateral DRG. Besides, the trauma to the uterus in the EM model might impact the pain behavior and signaling pathway in the DRG. In order to determine that the observed behavioral and molecular changes resulted from the EM-pathology, but not the excision of uterine horn tissue, we excided the uterine horn tissue without any transplant. Compared to the control group (opening the abdominal cavity only), the excision of uterine horn tissue did not induce mechanical pain in the musculus gastrocnemius (Fig. S2A). In addition, the excision of the uterine horn did not alter the expression of HMGB1, TLR4, MyD88, and p-NF-κB in the L3–L5 DRGs (Fig. S2B-C). The above results suggested that EM-pathology induced mechanical pain in the ectopic site with the activated TLR4-MyD88 pathway in the DRG.

### EM activated astrocytes and microglia in SDH

**Fig. 8:**
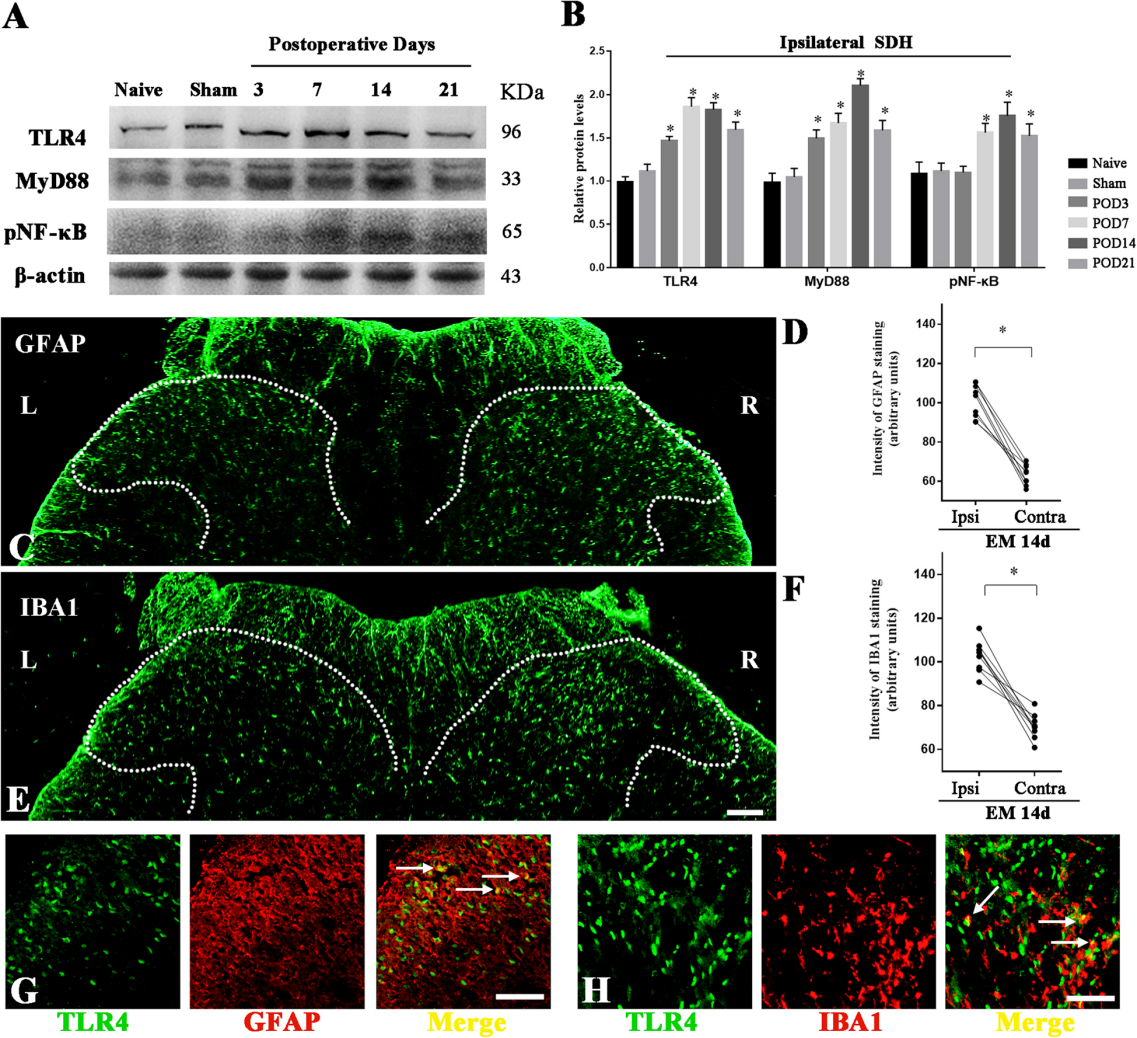
Activation of astrocytes and microglia in ipsilateral SDH of EM rats. **a** Western blot analyzed the time course of TLR4, MyD88, and pNF-κB-p65 expression in ipsilateral SDH after EM. **b** Quantification of protein levels for TLR4, MyD88, and pNF-κB-p65 expression in ipsilateral SDH. *N* = 4, *p < 0.05 versus naive and sham, one-way ANOVA followed by Bonferroni’s post hoc test. **c**–**f** Representative microscopic images of immunostaining for marker of astrocytes (**c**, GFAP) and microglia (**e**, IBA1) in SDH after EM. Scale bar 200 μm. SDH were indicated by dotted lines. Astrocytes and microglia were significantly activated in the ipsilateral SDH (right) compared to contralateral SDH (left). Fluorescence intensity was applied to measure the relative protein levels. Nine slices from 3 rats in each group, ^*^p < 0.05, Student’s *t* test. Ipsi: ipsilateral; Contra: contralateral. **g** Immunofluorescence staining showed the colocalization of TLR4 and GFAP in the SDH of EM 14d rats. Scale bar 100 μm. **h** Immunofluorescence staining showed the colocalization of TLR4 and IBA1 in the SDH of EM 14d rats. Scale bar 100 μm

It has been widely reported that TLR4 signaling evoked extensive activation of glia in the spinal dorsal horn (SDH), which was associated with multiple types of pain. Our previous study also showed that MyD88 was expressed in astrocytes and microglia and participated in neuropathic pain induction and persistence. In this study, western blotting showed that EM persistently upregulated the expression of TLR4 and MyD88 in the spinal dorsal horn from POD3. Increased phosphorylation of NF-κB p65 also indicated the activation of TLR4 signaling in the spinal dorsal horn of EM rats (Fig. 8a, b). GFAP is the marker for activated astrocytes in the CNS, while IBA1 is considered as an activated marker for activated microglia. Given the observed activation of astrocytes and microglia under multiple pain-related pathologies, we examined astrocytes (GFAP) and microglia (IBA1) activation by immunofluorescence staining in the SDH. Our immunofluorescence showed that astrocytes and microglia were both activated on ipsilateral (right) SDH compared to contralateral (left) SDH (Fig. 8c–f). In addition, the immunofluorescence staining showed the colocalization of TLR4 with GFAP and IBA1 in the SDH of EM rats (POD14), indicating that EM upregulated TLR4 in astrocyte and microglia (Fig. 8g, h).

### Blocking TLR4-MyD88 signaling pathway alleviated pain induced by EM

**Fig. 9:**
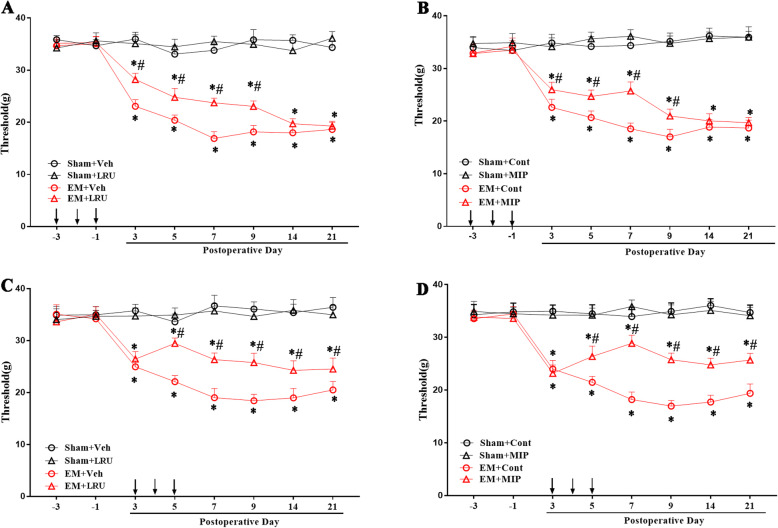
Attenuated mechanical pain by administration of LRU and MIP for EM rats. **a**, **b** Pre-intrathecal injection of LRU and MIP inhibited the development of EM-induced mechanical allodynia. Each administration was indicated by an arrow on the 3, 2, and 1 day before EM surgery. **c**, **d** Mechanical allodynia was alleviated by intrathecal injection of LRU and MIP in the early phase of EM operation. Each administration is indicated by an arrow on the 3, 4, and 5 days after EM surgery. LRU (20 μg) was administrated i.t. in a volume of 20 μl in endotoxin-free water (Veh). MIP (500 mM) was administrated i.t.in a volume of 20 μl. The control peptide was used in the control (Cont) group. *N* = 7 in each group, two-way ANOVA followed by Bonferroni’s post hoc test, *p < 0.05 EM + Veh versus sham + Veh and EM + LRU versus sham + LRU (for **a** and **c**); EM + Cont versus sham + Cont and EM + MIP versus sham + MIP (for **b** and **d**). ^#^*p < 0.05*, EM + LRU versus EM + Veh (for **a** and **c**) and EM + MIP versus EM + Cont (for **b** and **d**)

To measure the behavioral effects of TLR4 signaling on EM-related pain, we blocked the TLR4 signaling pharmacologically and observed behavioral changes. We used the specific TLR4 antagonist (LPS-RS-Ultra, LRU) and MyD88 homodimerization inhibitory peptide (MIP) to inhibit TLR4 and MyD88, respectively. The intrathecal injection was performed to deliver LRU (20 μg in 20 μL) or MIP (500 μM in 20 μl) into the lumbar cistern. First, drugs were daily administrated from − 3 to – 1 day before the surgery. Our data showed that pre-administration, both LRU and MIP, alleviated pain in EM rats from POD3 to POD9, while no effect was detected in sham rats. However, both the vehicle of LRU and the control peptide could not relieve the pain in EM rats (Fig. 9a, b). These results indicated that blocking of TLR4 signaling interfered with the development of pain in EM. Moreover, drugs were delivered intrathecally daily for three consecutive days from POD3. We found that both LRU and MIP administration relieved the EM-induced pain with a long-lasting alleviative effect from POD7 to POD21. The buffer for LRU and control peptide of MIP, administrated in the same manner, showed no effect (Fig. 9c, d).

### Blocking TLR4-MyD88 signaling pathway suppressed the activation of NF-κB in DRG and activation of glia in SDH under EM

**Fig. 10:**
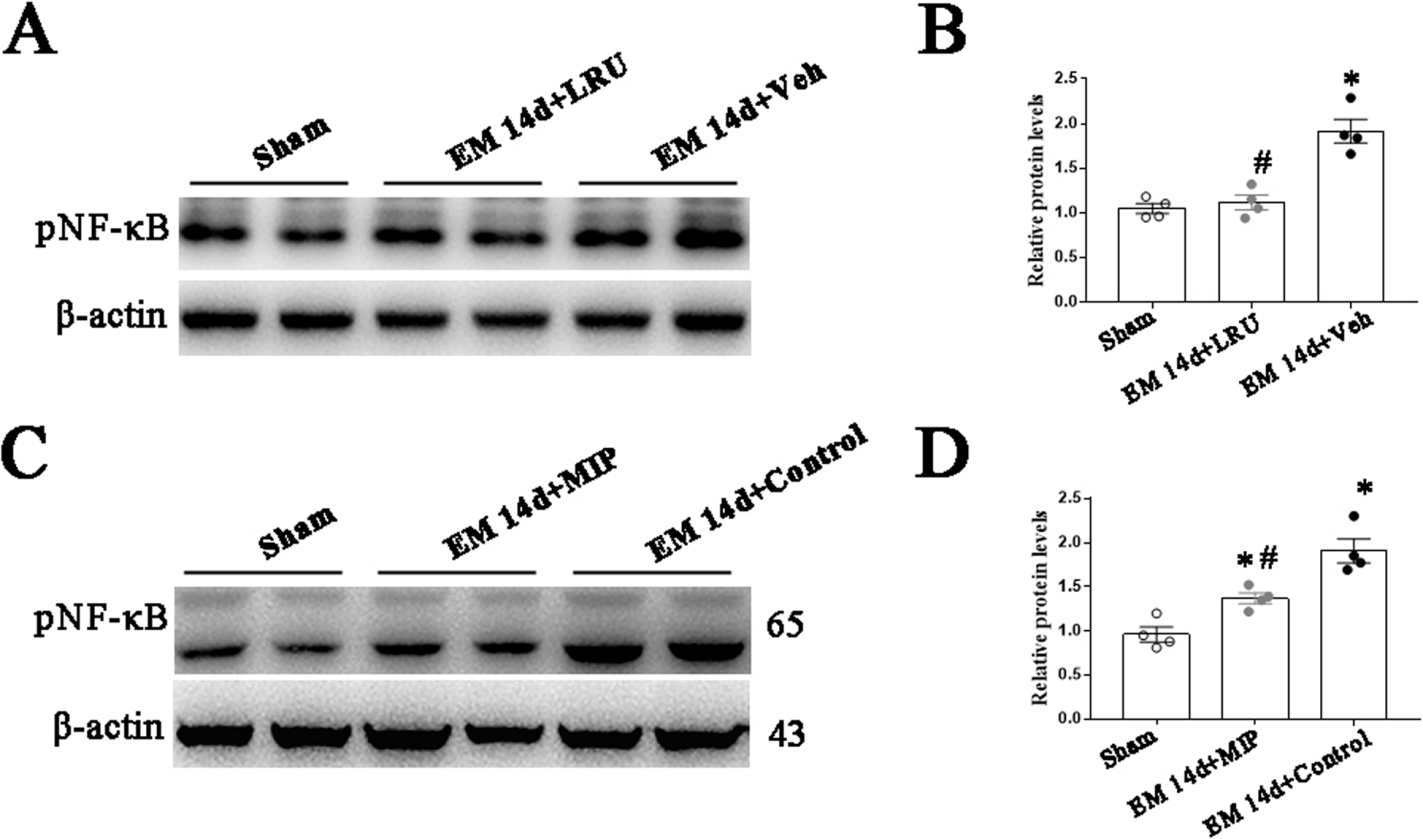
Intrathecal administration of LRU and MIP decreased pNF-κB-p65 protein expression in rat DRG after EM. **a** LRU or Vehicle was delivered intrathecally daily for three consecutive days from POD3. The representative protein bands of pNF-κB-p65 in DRG from sham rats, EM 14d + LRU rats and EM 14d + Vehicle rats. **b** LRU downregulated the expression of pNF-κB-p65 protein in the DRG induced by EM. *n* = 4, *p < 0.05, EM 14d + vehicle versus sham; ^#^*p < 0.05,* EM 14d + LRU versus EM 14d + vehicle, one-way ANOVA followed by Bonferroni’s post hoc test. **c** MIP or control peptide was delivered intrathecally daily for three consecutive days from POD3. The representative protein bands of pNF-κB-p65 in DRG from sham rats, EM 14d + MIP rats, and EM 14d + control rats. **d** MIP downregulated the expression of pNF-κB-p65 protein in the DRG induced by EM. *n* = 4, *p < 0.05, EM 14d + control versus sham and EM 14d + MIP versus sham; ^#^*p < 0.05*, EM 14d + MIP versus EM 14d + control, one-way ANOVA followed by Bonferroni’s post hoc test

To investigate the mechanism of pain alleviation by LRU and MIP, we examined the expression of phosphorylated NF-κB p65 in sensory neurons. Western blotting revealed that LRU, applied from POD3 to POD5, decreased the phosphorylation of NF-κB p65 in the DRG on POD14. Meanwhile, MIP treatment, from POD3 to POD5, also decreased the phosphorylation of NF-κB p65 in the DRG on POD14 (Fig. 10a–d).

**Fig. 11:**
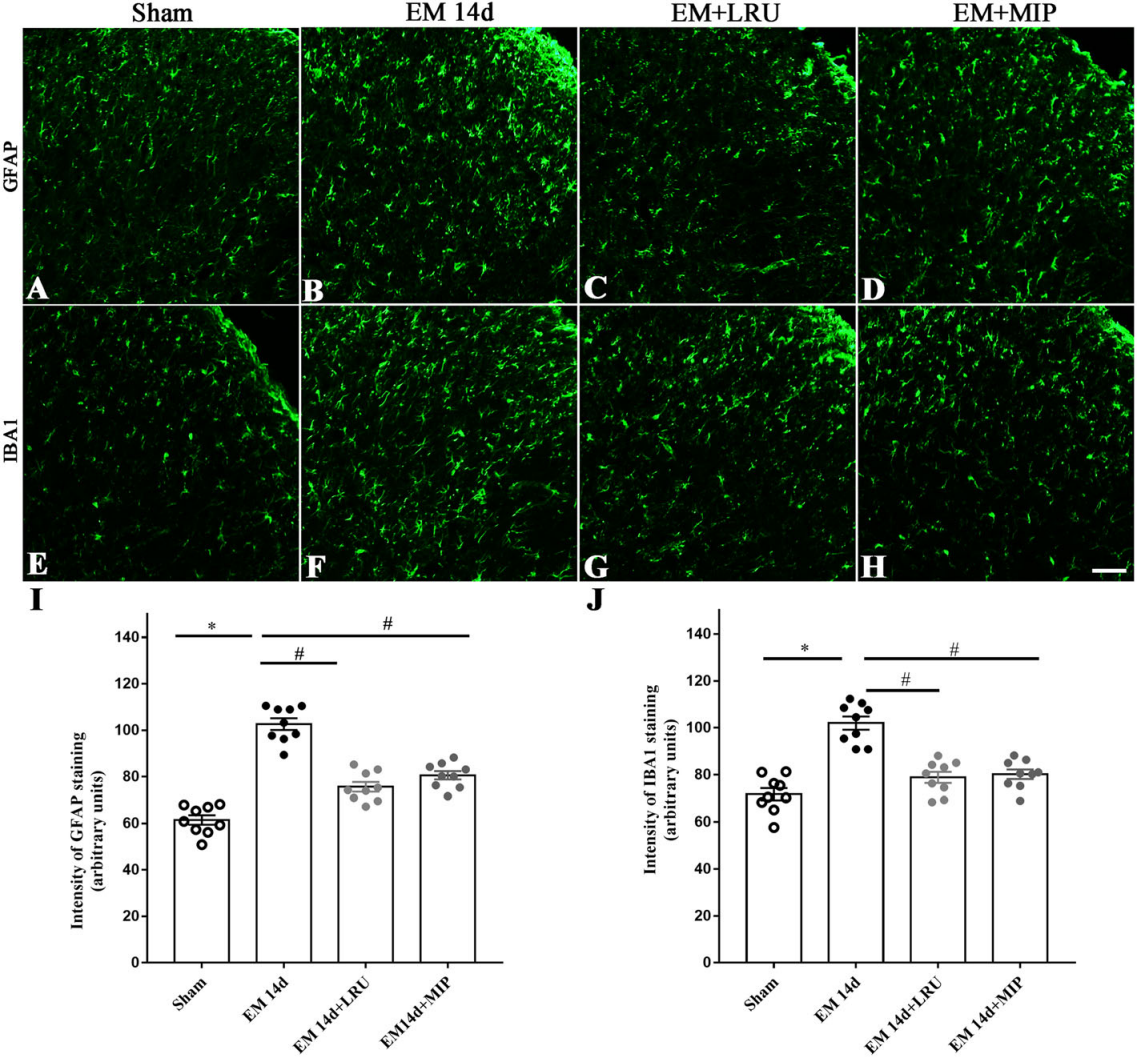
Attenuated activation of astrocyte and microglia in SDH of EM rats by both LRU and MIP. **a**–**d** Representative immunofluorescence images showed inhibitory effects of LRU and MIP on GFAP expression in the SDH of EM rats (14 days after surgery). **e**–**h** Immunofluorescence showed attenuated IBA1 expression in the SDH of EM rats (14 days after surgery) receiving LRU or MIP. **i**, **j** Quantitative analysis of fluorescence intensity showed that EM upregulated the expression of GFAP and IBA1 in the SDH. MIP and LRU attenuated the expression of GFAP and IBA1 in the SDH of EM rats. Nine slices from 3 rats in each group, *p < 0.05, EM 14d versus sham group; ^#^*p < 0.05*, EM 14d versus EM + LRU and EM 14d versus EM + MIP group, one-way ANOVA followed by Bonferroni’s post hoc test

Immunofluorescent staining was performed to determine the changes of glial activation after blocking TLR4 signaling in EM rats. Our results showed that both LRU and MIP treatment, applied from POD3 to POD5, decreased the expression of GFAP on POD14, indicating decreased activation of astrocyte (Fig. 11a–d, i). In addition, EM also induced the upregulation of IBA1 and microglial activation on POD14, which could be suppressed by intrathecal administration of LRU or MIP from POD3 to POD5 (Fig. 11e–h, j).

## Discussion

In this study, the rat model of endometriosis was performed successfully and exhibited hyperalgesia, determined by anatomical, pathological, and behavioral studies. Not only the implanted uterine tissue, but the DRG and SDH showed a significant upregulation of HMGB1. TLR4 and MyD88 were both upregulated in small-diameter nociceptive neurons in the DRG. Meanwhile, phospho-NF-κB p65, the responsible transcription factor of the TLR4-MyD88 dependent pathway, was upregulated in sensory neurons. We also detected the activation of astrocyte and microglia in the SDH after EM, which might trigger an inflammatory microenvironment and facilitate pain transmission in the SDH. Treatment with TLR4 antagonist (LRU) or MyD88 homodimerization inhibitory peptide (MIP) not only alleviated the hyperpathia in EM, but also reversed the phosphorylation of NF-κB p65 in the DRG and the activation of astrocytes and microglia in the SDH. These results suggested that the TLR4-MyD88 signaling pathway in the DRG and SDH, which might be activated by pro-inflammatory HMGB1, was involved in EM-induced pain.

EM, an estrogen-dependent disorder, is closely related to the estrous cycle, the hallmark symptom of which is chronic pain around the affected region [37]. Surgical induction of endometriosis was first established by Alvarez P. Measurement of hyperalgesia at the site of endometriosis lesion was performed together with the determination of the estrous cycle phase. In this model, pathological and behavioral changes were observed for a considerable period after surgery, regardless of their estrus cycle status. In our study, the EM pathology was determined by characteristic cystic lesions with a reddish-brown to amber fluid in endometriosis rats, which were consistent with previous studies [31, 32, 38]. In addition, HMGB1 was expressed in the transplanted tissue with chronic mechanical pain around the graft site. Previous studies about relieving pain in EM had focused on hormone and reproductive systems from the perspective of gynecology, but the efficacy has been unsatisfactory or with inevitable side effects [9]. Our present study focused on changes in the neuronal system and the neuro-immune communication in the TLR4-MyD88 signaling pathway, finding their possible role in the mechanism of hyperpathia in the rat EM model.

HMGB1, a nuclear non-histone DNA-binding protein, is ubiquitously expressed in multiple cell types, which can be released by necrotic cells during tissue injury in response to inflammatory signals. There are two forms of HMGB1: the nucleus form functions mainly as a transcriptional regulator, and the cytoplasmic form usually elicits the inflammatory process [39]. As a proinflammation mediator, HMGB1 was induced in the DRG and/or SDH in neuropathic conditions, which also evoked intense cytokines and involved in relative pain symptoms [24, 40]. This study verified that neuronal HMGB1 in the DRG and SDH was also induced in the EM model, especially the cytoplasmic HMGB1. The neuronal HMGB1 might be secreted by the relevant cells and elicit following neuroinflammatory cascades through its receptors, including TLR4.

The TLR family plays an essential role in innate immune responses, recognizing external pathogens and protecting host cells through opportune immunoreaction [41]. TLR4, one of the potent HMGB1 receptors, is expressed in DRG, TG, and SDH, facilitating pain and itch sensation [27, 41, 42]. In the DRG, TLR4 was mainly expressed in small-diameter nociceptors, which involved the sensitization of the TRPV1 channel and the persistence of chronic neuropathic pain [22]. TLR4 was also detected in microglia and astrocytes, regulating glial activation and spinal inflammatory microenvironment, which might be responsible for the central sensitization of pain [43, 44]. Challenged by heat shock protein 90 (HSP90), HMGB1 or LPS, TLR4 could activate both MyD88-dependent and MyD88-independent signaling pathway and mediate the allodynia and neuroinflammatory process [41]. MyD88, the adaptor protein for TLR4, also expressed in nociceptors and glial cells and induced glial activation and the production of TNF-α and IL-1β, which was related to neuropathic pain [34]. Our data proved the activation of the TLR4-Myd88-NF-κB pathway both in DRG and SDH, which might be responsible for the nociceptive facilitation in EM rats. Considering the special role of HMGB1 in the EM pathology, our experiments aimed to reveal the pair of HMGB1 and TLR4 in chronic EM pain. As the many-to-many relationship of the ligands and receptors, the effects of other pairs, such as HMGB1 and RAGE, could be further studied by knockout mice or multiple pharmacological designs.

As TLR4 is not the only receptor for HMGB1, and the TLR4 signaling is conducted by both MyD88-depended and MyD88-independent pathway, TLR4 and MyD88 were blocked respectively to observe the effect of TLR4-MyD88 pathway on EM-related pain. We performed pharmacological intervention by intrathecal injection to specifically disturb the TLR4-MyD88 pathway before or after the construction of EM pathology. Preemptively, intrathecal application of LRU or MIP could delay the occurrence of mechanical allodynia. The treatment of LRU or MIP after the formation of initial hyperpathia reversed the pain symptom and showed a long-lasting effect. Consistent with altered pain-related behavior, the phosphorylation of NF-κB-p65 in the DRG and the activation of glia in the SDH was also reversely changed by blocking TLR4-Myd88 signaling in EM rats. By this respective blocking of TLR4 and Myd88 adaptor, our data suggested that the activated TLR4-MyD88 signaling pathway in the DRG and SDH contributed to mechanical pain in EM rats.

## Conclusions

Our study demonstrated that endometriosis induced the expression of HMGB1 in the transplant, dorsal root ganglion, and spinal dorsal horn. Also, endometriosis upregulated the expression of TLR4 and Myd88 in small-diameter nociceptors with increased phosphorylation of NF-κB in the DRG. In addition, endometriosis-activated astrocytes and microglia with upregulated TLR4-Myd88 signaling in the spinal dorsal horn. Blockade of the TLR4-MyD88 signaling pathway attenuated mechanical hyperalgesia and reversed the molecular changes, which implicates this pathway in EM-related pain mechanisms. TLR4-MyD88 signaling pathway may play a vital role in EM-induced pain and serve as a potential therapeutic target.

## Supplementary Information

**Additional file 1: Fig. S1** Analysis of HMGB1 expression in DRG and SDH. A. Immunofluorescence staining showed EM significantly upregulated the cytoplasmic HMGB1 in the DRG. N=3, one-way ANOVA, *P < 0.05 versus sham. B. Percentages of cytoplasmic HMGB1-positive DRG neurons from EM 14d group in neurons with marker of CGRP, IB4 and TRPV1. C. Analysis of staining intensity for HMGB1 indicated the upregulation of HMGB1 by EM in the SDH. 9 slices from 3 rats in each group, *P < 0.05, Student's t-test, EM 14d versus Sham.

**Additional file 2: Fig. S2** The excision of uterus horn tissue did not induced mechanical pain at the gastrocnemius muscle. A. The time course of mechanical threshold at the gastrocnemius muscle for rats receiving the excision of uterus horn or not. N=8 in each group, Two-way ANOVA followed by Bonferroni's post-hoc test. B-C. The expression of HMGB1, TLR4, MyD88 and pNF-κB-p65 was not altered by the excision of uterus horn tissue. N=4, One-way ANOVA followed by Bonferroni's post-hoc test.

## Abbreviations

TLR4: Toll-like receptor 4
CGRP: Calcitonin gene-related peptide
CNS: Central nervous system
DRG: Dorsal root ganglion
EM: Endometriosis
GFAP: Glial fibrillary acidic protein
HMGB1: High mobility group box 1
IB4: Isolectin B4
IBA1: Allograft inflammatory factor 1
LRU: LPS-RS-Ultra
MIP: MyD88 homodimerization inhibitory peptide
Myd88: Myeloid differentiation primary response protein 88
NF-κB: Transcription factors of the nuclear factor κB
POD: Postoperative day
SDH: Spinal dorsal horn
