# Supplementary figures and images for: Suppression of TLR4-MyD88 signaling pathway attenuated chronic mechanical pain in a rat model of endometriosis
Source: J Neuroinflammation. 2021 Mar 5;18:65. doi: 10.1186/s12974-020-02066-y (PMC7934423; doi:10.1186/s12974-020-02066-y)

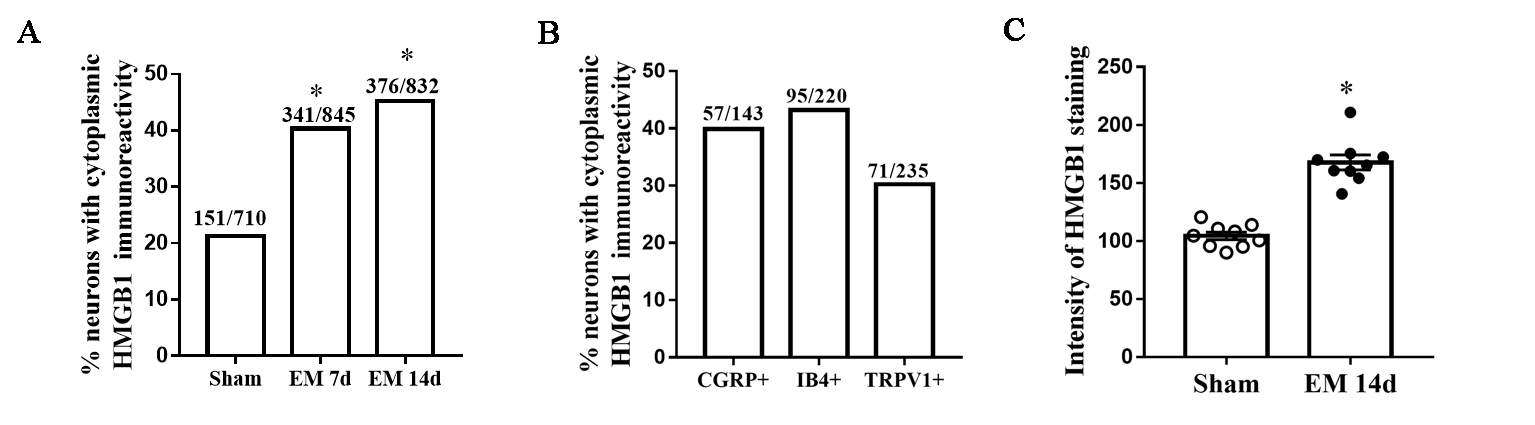

Supplement: Supplementary file 1 — Additional file 1: Fig. S1 Analysis of HMGB1 expression in DRG and SDH. A. Immunofluorescence staining showed EM significantly upregulated the cytoplasmic HMGB1 in the DRG. N=3, one-way ANOVA, *P < 0.05 versus sham. B. Percentages of cytoplasmic HMGB1-positive DRG neurons from EM 14d group in neurons with marker of CGRP, IB4 and TRPV1. C. Analysis of staining intensity for HMGB1 indicated the upregulation of HMGB1 by EM in the SDH. 9 slices from 3 rats in each group, *P < 0.05, Student's t-test, EM 14d versus Sham. [file 12974_2020_2066_MOESM1_ESM.jpg]

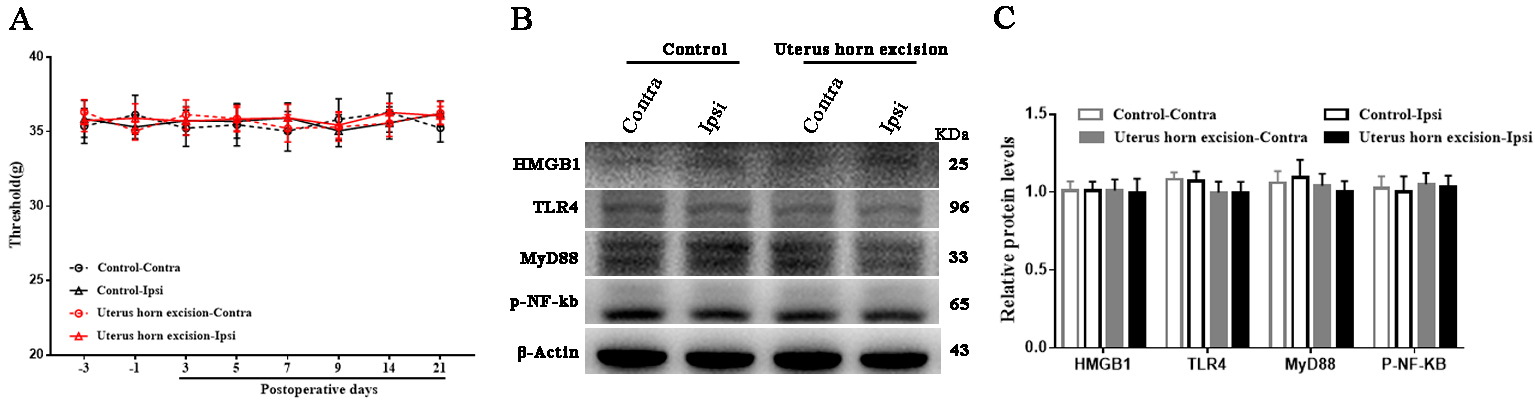

Supplement: Supplementary file 2 — Additional file 2: Fig. S2 The excision of uterus horn tissue did not induced mechanical pain at the gastrocnemius muscle. A. The time course of mechanical threshold at the gastrocnemius muscle for rats receiving the excision of uterus horn or not. N=8 in each group, Two-way ANOVA followed by Bonferroni's post-hoc test. B-C. The expression of HMGB1, TLR4, MyD88 and pNF-κB-p65 was not altered by the excision of uterus horn tissue. N=4, One-way ANOVA followed by Bonferroni's post-hoc test. [file 12974_2020_2066_MOESM2_ESM.jpg]
